# The Institutional Library

**Published:** 1918-12-07

**Authors:** 


### THE INSTITUTIONAL LIBRARY.

### 50,000 MILES ON A HOSPITAL SHIP.*

It is not difficult to realise the avidity with which the dozen budgets of which this volume is composed must have been read and re-read by the lady to whom her bachelor son first penned them. No man can write a record of his work and experiences without at the same time revealing himself in no inconsiderable measure; and whilst we meet "The Padre" as strangers, we part as acquaintances and friends. Having said so much, it is needless to emphasise the deep pleasure the perusal of this book has given us.

"The Padre" gives us, in narrative form, a running account of a series of voyages totalling over 50,000 miles and ranging from various English ports to Gibraltar, Marseilles, Valetta, Mudros, Lemnos, Salonika, the Dardanelles, and Alexandria. We are initiated into the general life on board, are introduced to many of his comrades, and are glad to find graceful tributes to "The Angels" (very human ones in cap and apron). We have before us glimpses of humour, pathos, pluck, cheerfulness, patience, and grit—a composite picture, and the wise will be content.

In the very nature of the case the main interest of the book lies in the author's record of his work amongst the men, and we feel assured that all who are really interested in this department of work amongst the boys, of whom none of us can think or write without a sense of pride and gratitude, will be drawn towards the Padre by reason of his tact, common sense, and adaptability in the face of unusual, if not unique, circumstances.

His common sense and adaptability are well exemplified in his attitude towards the questions of admitting Christian men of other denominations than his own to the Holy Communion; of a slavish adherence to the letter of the Prayer Book in the presence of men, to many of whom he would have been unintelligible had he done so; and united services in which ministers of various denominations took part. "The Padre" has much to say, as the result of his experiences, on the matter of Christianity after the war; and this book is emphatically one to be read by all who have these interests at heart. All such should acquaint themselves with what this Padre, in common with many others, tells us of his convictions and premonitions in regard to these matters.

* *50,000 Miles on a Hospital Ship.* By "The Padre." (London: Religious Tract Society. Price 3s. 6d. net.)

### THE SCIENCE OF PROGNOSIS.

In this, the second edition, of the latest recruit to the famous "Index" series,* a considerable amount of fresh material has been added, notably from experience and statistics rendered available by the immense growth of our knowledge of military surgery and orthopædics and diseases incidental to foreign campaigns. Thus amongst the freshly included material we find articles on Tetanus, Gas Gangrene, Septic Peritonitis, and Gunshot Wounds, whilst the various articles on tropical diseases have been largely rewritten.

Its value as a work of reference is, naturally, tremendously enhanced by the strict impartiality that is maintained. The general practitioner, swayed by the eloquence of the over-sanguine specialist, will find here his antidote, miraculous cures are given short shrift in the cold arrangement of the compiler's statistics.

The articles by Poynton on rheumatoid and osteoarthritis, for example, make refreshing reading. The various theories are briefly dealt with, toxæmic, nutritional, hereditary, and the rest, and the writer frankly admits that we are as unable to cure the disease as we are to explain its origin.

With typhoid fever, on the other hand, not only are we fully cognisant of the methods of production, but we can practically prevent an outbreak and by selected therapeutic measures reduce the mortality to almost half the normal ratio. Thus at the Brisbane General Hospital under the usual expectant treatment the mortality was 14.8 per cent., whilst under the cold and tepid bath treatment it fell to 7.5 per cent., and this reduction seems to be a fairly constant one all over the world. With vaccines Petrovitch, during the Serbian epidemic of 1915, claims to have reduced the mortality to 2.7 per cent. in a series of over two thousand cases. Liberal diet, too, seems to have a considerable influence in reducing the death-rate, for Coleman, of New York, records a mortality of 17.6 per cent. among patients restricted to milk as against 8.1 amongst those allowed a more liberal diet. The prophylactic inoculation against typhoid may rightly be claimed as one of the greatest triumphs of medicine. The statistics have been often quoted, but those for the United States Army may perhaps be new. Prior to its introduction the case rate per thousand varied between 3.1 in 1905 and 6.9 in 1902. Compulsory inoculation was introduced in 1910, and the following year the figure had fallen to .8, by 1913 it was as low as .1 per thousand, and at this level it has remained. The prognosis for syphilis under the newer methods of treatment is a question of considerable interest. At present, though it seems impossible to guarantee a cure in any instance, yet the number of patients showing relapses after treatment has been discontinued for twelve months (either a clinical relapse or a positive Wassermann without symptoms) if the disease is taken in the primary stage is only 11.9 per cent— *i.e.* more than seven-eighths are permanently cured. The substitutes placed on the market since the failure of the German supply are not by any means so effective. Ernest Lane considers that of these galyl is by far the most effective and that twelve injections will secure a negative reaction. But with all these preparations injections have to be continued for two years in combination with mercury before the patient can be discharged as cured.

This work is as it stands a remarkable achievement, of the highest value to every practitioner of medicine as well as to the student about to sit for his degree. Later editions will no doubt contain a fuller account of the prognosis for war injuries, upon which the work is at present incomplete.

* *An Index of Prognosis.* Edited by A. Rendle Short. (Bristol: Wright. Price 30s. net.)

**Institutional Library** (*continued*).

**Vaccines and Sera in Military and Civilian Practice** By A. Geoffrey Shera, Hon. Capt. R.A.M.C. (London: Henry Frowde, Oxford University Press; Hodder and Stoughton, Warwick Square, E.C. Price 7s. 6d. net.)

Here is much knowledge in a little space. The book is admirable. Treatment by vaccines and sera is still on trial, and as yet has hardly had fair trial. The extravagant claims of some, having been followed by the disappointments that always follow such, have led to an undeserved decrying by others. Captain Shera seeks to hold the balance true, to set forth what may reasonably be expected and possible developments Practical methods, minute directions, and statistics are given. Especially interesting is the account of the results in septic war wounds. In this branch of surgery much may be expected from the scientific use of these remedial measures and measures prophylactic. The chapters on dysentery are worth very careful study by any medical man likely to go to the East. The introduction by Sir Clifford Allbutt, K.C.B., commends the book, and most medical men will learn much from a careful reading of the really valuable matter that is in every chapter of it.

**Wise Parenthood: A Sequel to "Married Love.'** By the same Author, Marie Carmichael Stopes, D.Sc. (London: A. C. Fifield, 13 Clifford's Inn. Price 2s. 6d. net.)

The object of this book, which is a sequel to the author's "Married Love" (now, practically without advertisement, in its seventeenth thousand), is to describe the scientific and least unsatisfactory methods of birth control for healthy married people. It would be unfair to the author, and her readers, to read the sequel unless and until "Married Love" had been read. Our readers will remember the review which appeared in The Hospital of June 1, p. 183, and to them the present handbook needs little introduction. The practice of birth control is general. It has passed beyond the stage of discussion, and the arguments in its favour seem to have more weight than those which have proved impotent against it. Accepting the fact as inevitable, what is the best method æsthetically and scientifically? Dr. Stopes, like all who have been consulted on the matter, knows that the practice has grown up *in spite* of the dislike which it has aroused. But desperate people have offended their sensibilities rather than face disunion, or perennial pregnancy, or something worse—namely, the streets. To meet the immense æsthetic difficulty, Dr. Stopes' method has the great advantage of being applied long before the moment when it will be needed. The rite of marriage is uninterrupted by it. That is an immense gain. The method seems to have fewer objections than most, but it would be unfair to the author to describe it here. The arguments against the common practices are set forth with insight and care. An attempt to pith a small book would do the injustice of seeming to reduce it to the level of a list of directions. It is much more; but the method is that which people, including many doctors, want to know. The work is especially for those happy people who recognise the kindred duty and delight of having by a healthy mother healthy children, without whom no marriage is a right marriage, or complete. The wretches who wish to avoid all children are punished as only great offenders are punished, by the fulfilment of their desire. This little book, however, may show them the risks which they are incurring. It should also prove suggestive to husbands and wives who wish to use our slender stock of knowledge wisely.

**White and Martin's Genito-Urinary Surgery and Venereal Diseases.** By Edward Martin, A.M., M.D., F.A.C.S., John Rhea Barton Professor of Surgery, University of Pennsylvania; Benjamin A. Thomas, A.M., M.D., F.A.C.S., Professor of Genito-Urinary Surgery in the Polyclinic Hospital and College for Graduates in Medicine; Instructor in Surgery, University of Pennsylvania; and Stirling W. Moorhead, M.D., F.A.C.S., Assistant Surgeon to the Howard Hospital, Philadephia, Pennsylvania. Tenth Edition. (Philadelphia and London: J. B. Lippincott Company. Price 30s. net.)

In a noble volume replete with coloured plates and illustrations the Lippincott Company present the tenth edition of "White and Martin's Genito-Urinary Surgery and Venereal Diseases," now written by Edward Martin, A.M., M.D., F.A.C.S., Benjamin A. Thomas, A.M., M.D., F.A.C.S., both of Pennsylvania University, and Stirling W. Moorhead, M.D., F.A.C.S., of the Howard Hospital, Philadelphia. The previous edition was published in 1910, and the authors explain that the reason why the book has been allowed to remain out of print for so long has been not for any lack of enterprise and urging upon the part of the publishers, but because they have felt that the work must be reset, re-written, and re-illustrated to fairly and yet succinctly present the views and practices of to-day. In carrying out their task they have, as stated in their preface, incorporated in the text a brief but practical presentation of vaccines and serums; tests of renal function which are found most serviceable in estimating operative risks; high-frequency desiccation; laboratory diagnosis of syphilis and control of treatment; the accepted conservative and radical treatment cf prostatic hypertrophy, including those measures which have done so much to lower mortality, and have endeavoured fully to present those therapeutic methods which have received the general approval of the clinically experienced.

The arrangement of the text, which extends over some 900 pages, divided into 44 chapters, is perhaps a little unfamiliar to English readers, but in any case there must always be considerable difficulty in this connection when it is intended to discuss surgical diseases of the genito-urinary apparatus in view of the special importance of venereal disorders. Syphilology verily to-day constitutes a speciality within a speciality, and probably most practitioners would prefer to read what purports to be an exhaustive account of the etiology, pathology, and treatment of syphilis between the covers of a separate volume. In any case the arrangement of chapters has the merit of being thoroughly practical, the first five being entirely devoted to the clinical and laboratory examinations necessary for the accurate diagnosis of diseases primarily manifested in abnormalities of the genito-urinary system. Detailed examination of the urine, methods of determining kidney function, as well as the technique of urethroscopy and cystoscopy, are in turn discussed, a special chapter being given to the choice, care, and sterilisation of instruments.

Thus informed as to the equipment and knowledge necessary to investigate with scientific precision the signs and symptoms of maladies affecting the particular system under consideration, the general practitioner or senior student is able to proceed with the consideration of clinical details, and it is satisfactory to note that such common troubles as suppression, retention, and incontinence of urine are dealt with in the first chapter on clinical matters. There are few things of more importance

**The Institutional Library**—(*continued*).

to a medical man in general practice than a proper understanding of the uses of the catheter. Many a young doctor has been brought up sharp in early days of private practice by some problem which he could readily have solved had he possessed a right knowledge of this important little instrument. The description and pictures in "White and Martin" will certainly help to elucidate many points in this regard, but one would prefer to have found a far more detailed account from the general practitioner's point of view of when, why, and how to use a catheter. The special difficulties of the patient suffering from an enlarged prostate gland, who is advised to use a catheter himself regularly, scarcely receive enough consideration, although it is wisely pointed out that, admitting that habitual self-catheterisation is troublesome, nevertheless thoroughness in carrying out antiseptic details should not be sacrificed to convenience.

In a work of this kind the anatomy and surgery of the bladder naturally bulk largely, and we find four chapters devoted to this part of the subject, although one might well expect it to occupy more than the one-ninth of the book which it approximately does. The last twelve chapters deal with syphilis in all its many manifestations, and this part of the book takes us at times well away from the genito-urinary system into considerations of the effects wrought by the *Spirochœta pallida* in various parts of the body. It is this unavoidable digression from the main theme that makes it doubtful whether venereal diseases should ever be exhaustively dealt with in a book primarily dealing with genito-urinary diseases. It is also doubtful if the authors were wise in extending their survey so far as to include the subject of "psychopathic sexualis," which certainly can never be efficiently discussed within the limits of ten pages.

### AN ILL-CHOSEN EPITHET.

DR. HYSLOP, in his recommendation printed on the jacket of this book,* speaks of the moderation with which the author has treated the various subjects it contains. The epithet is ill chosen. Dr. Weatherly expresses without much moderation his spite against that one of the Commissioners of the Board of Control who is a lady, and of his own achievements he speaks without any moderation at all. Dr. Weatherly must know that Miss Dendy is precluded by her official position from taking any notice of his attacks, which, as made in print, and in a book, by a man against a lady, are, as far as our experience goes, quite unparalleled. Miss Dendy has, of course, the satisfaction of knowing that it is much more reputable to be attacked by Dr. Weatherly than to be praised by him, but though he does thus incidentally add to her high reputation, his intention was the reverse. The taste with which the book is written may be judged by this example. It is the most—well, autobiographical book we have ever perused, and contains more repetitions of the first personal pronoun than probably any other book in any language. If there is a page without several capital "I's" in it we have not found it, and the average number to a page seems to be about a dozen. The chief lessons inculcated by the book are that Dr. Weatherly has been consulted a great many times, by a great many people; that he is quite fearless in giving his opinion, which, though usually in opposition to that of other people, is always right; that he is on terms of friendship with a great many more or less distinguished men; that he is very successful in the treatment of his patients; that he is very much annoyed by "wretched red tape"; that if his advice were followed, things would be much better than they are; and that he is altogether a very fine fellow. With this impression on our minds, it is with some surprise that we find that the Medico-Psychological Association, of which Dr. Weatherly seems to be a distinguished member, refused to accept a report which he, or a committee of which he was chairman, presented to the Association; that though he has been urging for twenty-seven years that a defect in the law should be remedied, his voice has made no impression; and that so many judges have disregarded his evidence. It would almost appear as if other people were insensible to his many merits.

* *A Plea for the Insane.* By L. A. Weatherley, M.D. (London: Grant Richards, Ltd. 1918. 9 × 5¾. Pp. 238. Price 10s. 6d. net.)

**The Soldier's First Aid.** By R. C. WOOD, Q.M.S., Army Medical Corps. (London: Macmillan and Co. Price 2s. 6d. net.)

QUARTERMASTER-SERGEANT R. C. WOOD has been instructing the Canadians in first aid. His experience as a teacher has taught him just what the learner wants put before him. He has produced an excellent little book on first aid for all the emergencies of war. A few men in each platoon who had thoroughly mastered the teaching of this book would be of inestimable advantage to their wounded comrades. The book is well got up, is profusely illustrated by excellent photographs, and is written in a more pleasant and readable style than most of the treatises on first aid. Naturally in a very elementary manual such as this there is nothing new, but all in it is sound, and it can easily be carried in a soldier's pocket.

**Manuals of Health. The Baby.** By SOPHIA SEEKINGS, M.D., B.S., D.P.H. (London: Society for Promoting Christian Knowledge. Price 9d. net.)

MANY of the books and booklets produced on medical subjects, especially on infant care, raise the question, "For whom is this intended?" The little volume entitled "The Baby," by Dr. Sophia Seekings, published as the second of a series of "Manuals of Health" by the S.P.C.K., contains many hints, explanations, and admonitions of the kind urgently required by the poorest and least educated class of mothers. Unfortunately, its price is the practically prohibitive one of 9d. At half this cost, or less, the little book might have been sold at Mothers' Meetings of various types. Even in this case the language is not always simple enough. A labourer's wife does not grasp without explanation such words as *approximate*, *broncho-pneumonia*, *eruption of teeth*, and *mastication*, which last one friend of the present writer prefers in the form of "domestication" of her food.

It is a pity, for the matter is generally excellent. We may especially commend some less-used counsels, such as the desirability of quieting and not fussing and showing-off a precocious child, the need of early care in measles, the wisdom of teaching a baby to sleep with arms folded. A sensible woman giving addresses to gatherings of women would find this an excellent text-book for one such occasion, or even for two.

